# Metabolic Syndrome Is Associated with Oxidative Stress and Proinflammatory State

**DOI:** 10.3390/antiox9030236

**Published:** 2020-03-12

**Authors:** Margalida Monserrat-Mesquida, Magdalena Quetglas-Llabrés, Xavier Capó, Cristina Bouzas, David Mateos, Antoni Pons, Josep A. Tur, Antoni Sureda

**Affiliations:** Research Group in Community Nutrition and Oxidative Stress, University of the Balearic Islands, Health Research Institute of Balearic Islands (IdISBa), and CIBEROBN (Physiopathology of Obesity and Nutrition), E-07122 Palma, Balearic Islands, Spain; margalida.monserrat@uib.es (M.M.-M.); m.quetglas@uib.es (M.Q.-L.); xavier.capo@uib.es (X.C.); cristinabouvel@gmail.com (C.B.); davidfrom13@gmail.com (D.M.); antonipons@uib.es (A.P.); antoni.sureda@uib.es (A.S.)

**Keywords:** metabolic syndrome, oxidative stress, cytokine, inflammation, PBMCs

## Abstract

Metabolic syndrome (MetS) is associated with increased risk of developing diabetes and cardiovascular diseases. MetS is also characterized by an increase of oxidative stress which contributes to impaired inflammation, vascular function, and atherosclerosis. The aim was to assess the oxidative stress and inflammatory markers in plasma and PBMCs in adults with or without MetS. Antioxidant and inflammatory parameters were measured in peripheral blood mononuclear cells (PBMCs) of 80 men and 80 women over 55 to 80-years-old residing in the Balearic Islands without previously documented cardiovascular disease. Circulating leukocytes, neutrophils, lymphocytes, basophils, and monocytes were higher in MetS subjects with respect to those without MetS. Plasma levels of malondialdehyde, tumor necrosis factor α (TNFα), and interleukin 6 (IL-6) levels were higher in MetS subjects in both genders, but the superoxide dismutase activity was lower. The myeloperoxidase plasma activity was higher in the MetS male subjects. Higher activities and protein levels of catalase and glutathione reductase in PBMCs were observed in MetS subjects in both genders. Obtained data show that MetS is associated with oxidative stress and a proinflammatory state and with high antioxidant defenses in PBMCs probably derived from a pre-activation state of immune cells.

## 1. Introduction

The metabolic syndrome (MetS) comprises a cluster of visceral adiposity and high risk of developing diabetes, cardiovascular diseases (CVD), and mortality [1]. The prevalence of MetS has been increased over the last years due to the high degree of sedentary lifestyle of the population in general, which has become one of the major public health problems [2]. Moreover, the prevalence of MetS is higher in women than in men, specially over the age of 65. The cause is related to changes induced by menopause, with a tendency to increase abdominal adiposity and to develop insulin resistance and dyslipidaemia due to oestrogen deficiency [3,4]. 

MetS is characterized by an increase in oxidative stress and inflammation. Although the pathogenesis of MetS is very complex and not yet fully elucidated, it has been suggested that a prooxidant/antioxidant imbalance may play an important role in its development [5]. An excessive production of reactive oxygen (ROS) and nitrogen (RNS) species can react with virtually all biomolecules leading to oxidative damage [6,7]. Several studies have observed that patients with MetS have lower activities of antioxidant enzymes in plasma and higher levels of oxidative damage markers, mainly lipid peroxidation, compared to healthy patients, which may contribute to the establishment of an oxidative stress situation [8]. This increase in oxidative stress can contribute to the pathogenesis of MetS, favoring the appearance of inflammation, thrombosis, and atherosclerosis, altering vascular function and leading to vascular disease [9,10].

Previous studies have revealed a close relationship between the pathogenesis of obesity with the activation of the innate immune system [11,12]. In this sense, obesity is related to a state of low grade inflammation which, in turn, may be a causative role in generation insulin resistance, defective insulin secretion, and disruption of other aspects of energy homeostasis [12]. 

The central mechanisms underlying the pathophysiology of insulin resistance and MetS is a chronic state of inflammation [13,14]. The major risk factor responsible for the insulin resistance and the pathophysiology link to MetS is visceral adiposity, which is related to a diet containing high saturated fat, excess of calories and glucose content, and physical inactivity [15].

The production of adipocytokines including adiponectin, irisin, monocyte chemoattractant protein-1 (MCP-1), tumor necrosis factor-α (TNF-α), leptin, interleukin-6 (IL-6) are found to be deregulated in MetS due to the oxidative stress situation [16,17]. Moreover, inflammatory markers that allow monitoring of obesity and MetS include proinflammatory cytokines production such as TNF-α, IL-1β, and IL-6, which are mainly produced by the macrophages infiltration induced by obesity in the adipose tissue [18]. These proinflammatory markers are positively correlated with insulin resistance and the features of MetS [19].

Peripheral blood mononuclear cells (PBMCs) from subjects with stage 2 obesity have been reported to produce significantly less IL-2 (necessary for the generation of T-lymphocyte response) after phytohaemagglutinin (PHA) stimulation than PBMCs from nonobese subjects [20]. In addition, previous studies have shown an increase of IL-6 and TNFα production in human monocytes incubated with glucose [21], moreover glucose to PBMCs reduced IL-2, IL-6, and IL-10 production and simultaneously inhibited cell proliferation [22]. Altogether, the factors involved in the establishment of MetS are related to a proinflammatory and oxidative stress state and a deregulation of the innate immune system. 

The aim of the present study was to assess the oxidative stress and inflammatory markers in plasma and PBMCs in adults with MetS and without MetS.

## 2. Materials and Methods 

### 2.1. Design and Participants

A total of 160 adults (50% women) over 55 to 80 years-old residing in the Balearic Islands without documented CVD were included in the study. Half of the patients selected were considered as inclusion criteria, and the remaining patients were included if they met three or more of the following five criteria of metabolic syndrome: (1) Triglycerides ≥150 mg/dL; (2) HDL-cholesterol <40 mg/dL in men and <50 mg/dL in women; (3) blood pressure ≥130/85 mmHg; (4) fasting serum glucose level ≥100 mg/dL; (5) waist circumference ≥90 cm in men and ≥80 cm in women, according to the updated harmonized definition of the International Diabetes Federation and the American heart Association and National Heart, Lung, and Blood Institute [23]. Treatment with antidiabetic or antihypertensive agents was considered as a positive variable. Patients who did not meet some of the inclusion criteria or had some of the following criteria were excluded: Patients institutionalized, suffering from any physical or mental illness limiting their ability to respond to questionnaires, chronic alcoholism or drug addiction, and intake of drugs for clinical research over the past year. 

The study protocols followed the Declaration of Helsinki ethical standards and all the procedures were approved by the Ethics Committee of Research of Balearic Islands (refs. CEIC-IB2251/14PI and CEIC-IB1295/09PI). All participants were informed of the purpose and the implications of the study, and informed consent was obtained from all subjects. 

### 2.2. Anthropometric and Physical Activity Characterization

Anthropometric variables were performed by professional observers to minimize the inter-observer coefficients of variation. Height and weight were determined using a wall-mounted stadiometer and with high-quality electronic calibrates scales, respectively. Height was measured with a mobile anthropometer (Seca 214, SECA Deutschland, Hamburg, Germany) to the nearest millimeter, with the patient’s head maintained in the Frankfort Horizontal Plane position. Body weight and body fat were determined using a Segmental Body Composition Analyzer according to the fabricator’s protocol (Tanita BC-418, Tanita, Tokyo, Japan). The subjects were weighed in bare feet and light clothes, so 0.6 kg was subtracted for their clothing. Weight and height measures were used to calculate body mass index (BMI), specifically weight in kilograms divided by the square of height in meters. Blood pressure was measured in triplicate with a validated semi-automatic oscillometer (Omron HEM, 705CP, Hoofddrop, The Netherlands) in a seated position. Waist circumference (WC) was measured in duplicate, halfway between the last rib and the iliac crest by using an anthropometric tape. Waist-to-height ratio (WHtR) as cardiovascular risk biomarker, was calculated from waist (cm) divided by height (cm). The metabolic equivalents (METs) of the physical activity performed were calculated considering the rate of energy expenditure defined according to current knowledge [24]. The participants estimated the duration of the activities performed in min/week.

### 2.3. Blood Collection and Analysis

Blood samples were collected after an overnight fasting and biochemical analyses were carried out on fasting plasma glucose, total cholesterol, HDL-cholesterol, and triglycerides concentrations in local laboratories using standard enzymatic methods. Hematological parameters and cell counts were analyzed in an automatic flow cytometer analyzer Technion H2 (Bayer) VCS system. Patients were classified as “with MetS” (n = 80) and “without MetS” (n = 80) as described above [15].

### 2.4. Cell Isolation and Cell Viability Test

Venous blood samples of the participants in the study were assembled after 12 h of overnight fasted conditions from the antecubital vein in suitable vacutainers with EDTA as anticoagulant. The PBMCs fraction was purified from fresh whole blood and isolated following the protocol Separation of White Blood Cells [25] described before, using the reagent Ficoll-Paque PLUS (GE Healthcare Bio-Sciences AB, Uppsala, Sweden) [26,27]. Concisely, 6 mL of blood was introduced on 4 mL of Ficoll (proportion 1.5:1) and was then centrifuged at 900× *g*, for 30 min at 4 °C. Then, the plasma and the Ficoll phases were discarded and PBMCs layer was washed with phosphate-buffered saline (PBS) at pH 7.4. After that, it was centrifugated at 900× *g*, for 10 min at 4 °C. Plasma was obtained by centrifuging whole fresh blood at 1700× *g* for 15 min at 4 °C.

### 2.5. Protein Levels

Antioxidant enzyme protein levels of catalase (CAT), manganese superoxide dismutase (MnSOD), glutathione peroxidase (GPX), and glutathione reductase (GRd) in PBMCs were determined by Western Blot analysis. Cells were lysed with 200 μL of RIPA buffer [250 mM Tris/HCl, pH 8.0, 4.4% NaCl, 5% IGEPAL^®^, 2.5% deoxycholic acid, 0.5% sodium dodecyl sulfate (SDS)]. Fifteen-microgram protein of total cell extract was loaded in each lane of an SDS polyacrylamide gel of 15% acrylamide. The electrophoresis was at 60 V for 15–20 min and then 150 V for 1 h, the molecular weight marker used was Precision Plus Protein Kaleidoscope^TM^ (Bio-Rad). Bans were electrotransferred onto a midi format 0.2 μm nitrocellulose membrane by using Trans-Blot^®^ Turbo^TM^ Transfer System (Bio-Rad, Segrate, Milan, Italy). The membranes were blocked (5% nonfat powdered milk in TBS pH 7.5, containing 0.1% Tween 20) for 2 h and incubated with the corresponding primary monoclonal antibody over night at 4 °C in shaking (30 rpm). Antibodies anti-CAT (1:1000, rabbit) and anti-MnSOD (1:1000, sheep) were supplied by Calbiochem (Merck KGaA, Darmstadt, Alemania); anti-GRd (1:1000, mouse) and anti-GPx (1:200, goat) were supplied by Santa Cruz Biotechnology (Santa Cruz, CA, USA). Then, blots were incubated with a secondary peroxidase-conjugated antibody (1:5000) against specific primary antibody for anti-CAT, anti-MnSOD, and anti-GRd but for anti-GPx (1:10,000). Development of immunoblots was performed using an enhanced chemiluminescence kit (Immun-Star^®^ Western C^®^ Kit reagent, Bio-Rad Laboratories, Hercules, CA, USA). Protein bans were visualized and quantificated using the image analysis program Quantity One (Bio-Rad Laboratories). 

### 2.6. Enzymatic Determinations 

CAT activity in plasma and PBMCs was determined by the spectrophotometric method of Aebi based on the decomposition of H_2_O_2_ [28]. Superoxide dismutase (SOD) activity was measured in plasma and PBMCs by an adaptation of the method of McCord and Fridovich [29]. GRd activity was measured in PBMCs by an adaptation of the Goldberg and Spooner spectrophotometric method [30]. GPx activity was determined in PMBCs using a modification of the spectrophotometric method of Glohé and Gunzler [31]. Myeloperoxidase (MPO) was measured in plasma by guaiacol oxidation by monitoring the resultant tetraguaiacol compound at 470 nm [32]. All activities were determined with a Shimadzu UV-2100 spectrophotometer (Shimadzu Corporation, Kyoto, Japan) at 37 °C.

### 2.7. Malondialdehyde Assay 

Malondialdehyde (MDA) was determined in plasma by a specific colorimetric assay kit (Sigma-Aldrich Merck^®^, St. Louis, MO, USA) following the manufacturer’s instructions. The method is based on the reaction of MDA with a chromogenic reagent generating a stable chromophore. Briefly, plasma samples or standards were introduced in glass tubes containing n-methyl-2-phenylindole in acetonitrile: Methanol (3:1) mixture. HCl (12N) was added and samples were incubated at 45 °C for 1 h. The absorbance was measured at 586 nm and the MDA concentration was calculated with a standard curve of known concentrations. 

### 2.8. Cytokines Assay

Cytokine (TNFα and IL-6) levels were determined in plasma using individual ELISA kits (Diaclone, Besancon Cedex, France) following the manufacturer’s instructions to use. The overall intra-assay coefficient of variation was calculated to be 3.2% for TNFα and 4.4% for IL-6; the calculated overall inter-assay coefficient of variation was 10.9% for TNFα and 9.1% for IL-6. 

### 2.9. Statistics

Statistical analysis was performed using the Statistical Package for Social Sciences (SPSS v.25 for Windows, IBM Software Group, Chicago, IL, USA). Results are expressed as the mean ± standard error (SEM), and the level of significance was established at *p* < 0.05 for all statistics. Normality of data was assessed using the Kolmogorov–Smirnov test. The statistical significance of the data was checked by two-way analysis of variance (ANOVA) after adjustment for gender (G) and Metabolic Syndrome (MetS). The sets of data in which there was a significant MetSxG interaction were tested by one-way ANOVA. When significant differences were found between groups, a Bonferroni post hoc test was carried out. Women without MetS were taken as a reference group and referred to as 1. 

## 3. Results

### 3.1. Anthropometric and Hematological Parameters

The anthropometric characteristics of the participants in the study stratified by gender are shown in Table 1.

Men showed higher values in weight, height, and abdominal adiposity with respect to women. Weight, BMI, glucose, triglycerides, abdominal obesity, and WHtR were higher in both genders in the MetS groups, whereas HDL-cholesterol was lower. No significant differences were evidenced in systolic blood pressure, whereas diastolic blood pressure was lower in the MetS groups. Total physical activity measured as MET·hour/week was lower in the MetS groups, although only in women there were significant differences.

Hematological parameters of participants are shown in Table 2. No differences were reported in haematocrit and erythrocyte counts between MetS and without MetS groups, although both parameters were higher in men compared to women in the group without MetS. The number of leukocytes, neutrophils, lymphocytes, basophils, and monocytes were higher in patients with MetS with respect to the group without MetS, but no differences were evidenced in the number of eosinophils. Neutrophil counts were also higher in men with respect to women in the group without MetS.

### 3.2. Oxidative Stress Biomarkers

The results of enzymatic activities of CAT, SOD, and MPO in plasma and CAT, GRd, GPx, and SOD in PBMCs are shown in Table 3. The plasma SOD activity was lower in subjects with MetS. The MPO plasma activity was higher in men with MetS in respect to those without MetS, but maintaining similar values between women with and without MetS. In PBMCs, CAT and GRd activities were higher in patients with MetS in respect to those without MetS. No differences were found in PBMCs’ GPx and SOD activities. Significant differences between participants with and without MetS were found for CAT and GRd protein levels (Table 3), with higher levels in MetS groups. No differences were evidenced in GPx and MnSOD protein levels. No significant differences between genders were observed in any of the parameters analyzed.

**Figure 1:**
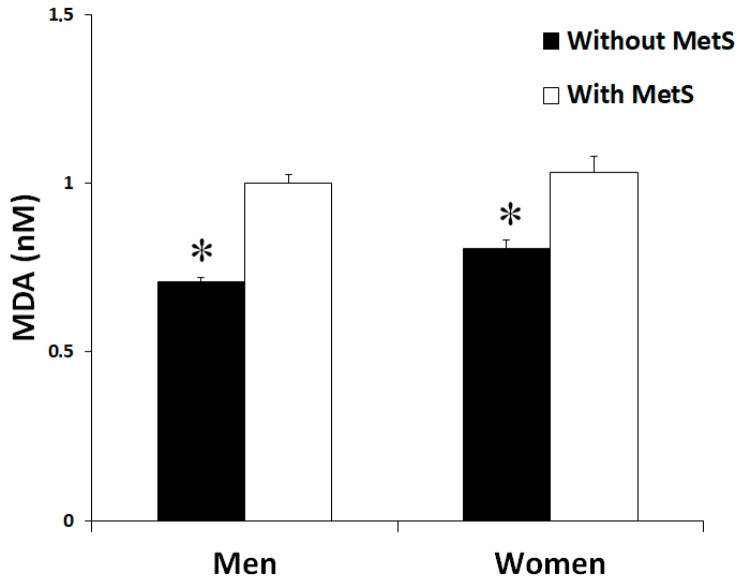
Malondialdehyde (MDA) levels in plasma men and women in the study, classified according to the metabolic syndrome. Significant effects of metabolic syndrome were found. Statistics: Two-way ANOVA. Results are presented as mean ± SEM. *Differences in means between participants without and with MetS.

Plasma MDA levels (Figure 1), as lipid peroxidation marker, reported significant differences between groups. Patients without MetS evidenced lower levels of MDA when compared with patients with MetS. No differences were reported between genders.

### 3.3. Cytokine Levels

**Figure 2:**
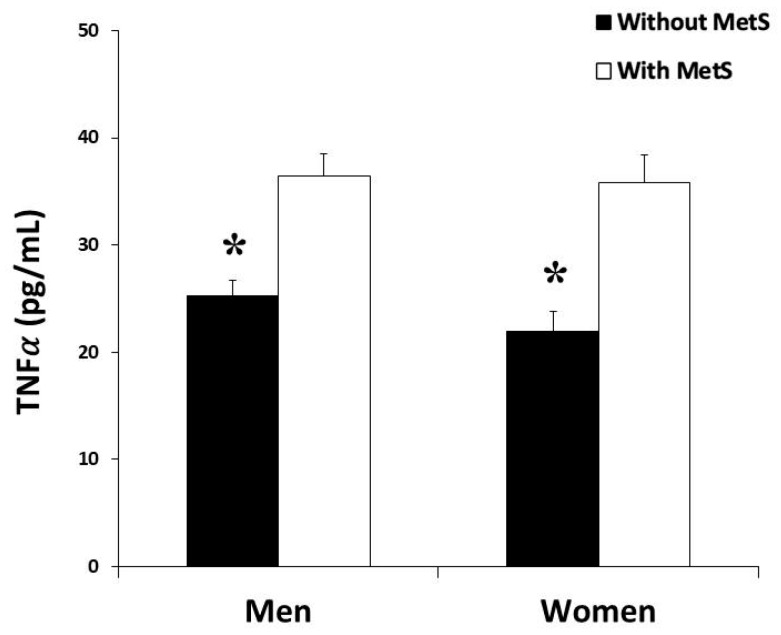
Tumor necrosis factor α (TNF𝛼) production rate in plasma men and women in the study, classified according to the metabolic syndrome. Results are presented as mean ± SEM. Significant effects of metabolic syndrome were found in both groups by two-way ANOVA. *Differences in means between participants without and with MetS.

**Figure 3:**
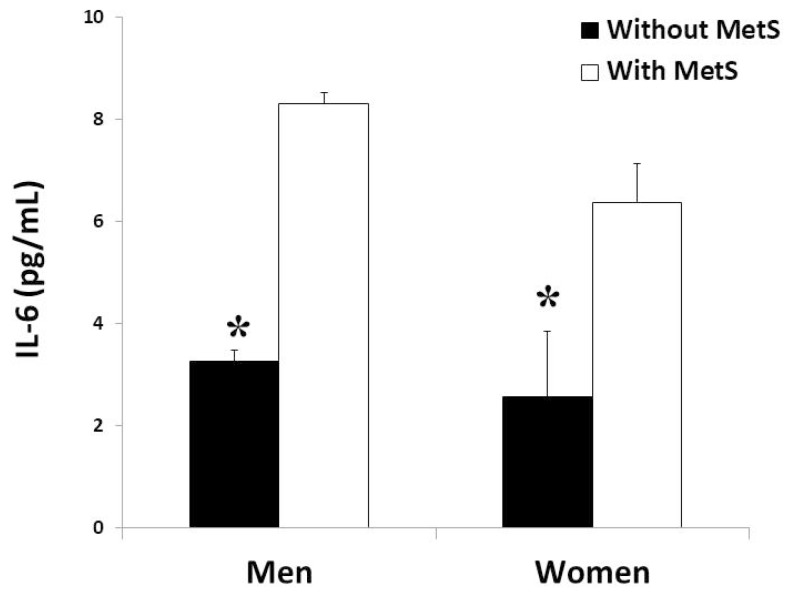
Interleukin 6 (IL-6) production rate in plasma (men and women) in the study, classified according to metabolic syndrome. Results are presented as mean ± SEM. Significant effects of metabolic syndrome were found in both groups by two-way ANOVA. *Differences in means between participants without and with MetS.

Plasma levels of the proinflammatory cytokines TNFα and IL6 were shown in Figure 2 and Figure 3, respectively. The levels of TNFα and IL-6 were significantly higher in the groups with MetS in both men and women, but without differences between genders.

## 4. Discussion

MetS is a growing concern in developed and developing countries directly related to CDV, cardiovascular mortality, and T2DM. MetS risk factors include central obesity, glucose intolerance, hyperinsulinemia, low HDL-cholesterol, high triglycerides, and hypertension [33]. Our results revealed that patients from both genders with MetS had altered anthropometric parameters and blood biochemical profile with respect to patients without MetS. Specifically, MetS patients presented higher body weight, BMI, abdominal obesity, WHtR, levels of glucose and triglycerides, and lower HDL-cholesterol. These alterations have been reported to be associated with unbalanced nutritional status, high cholesterol, animal proteins, simple carbohydrates, saturated fatty acid levels, and low fibre, vegetable proteins, monounsaturated and polyunsaturated fatty acids and complex carbohydrates consumption, and a sedentary lifestyle [34,35]. The lack of differences between groups with respect to systolic blood pressure and the presence of lower diastolic values in the group with MetS could derive from the fact that 71% of women and 80% of men with MetS take antihypertensive drugs.

Regarding blood immune cells, higher number in leukocytes, neutrophils, monocytes, and basophils has been observed in patients with MetS in both genders that can be related to the proinflammatory state associated with this disorder. In this sense, it has been reported that obesity causes adipose tissue inflammation and may contribute to release proinflammatory mediators and mobilization of immune cells. These results are in accordance with previous studies which suggested that adipose tissue could be a significant contributor to increased systemic inflammation in overweight and obese people [36,37]. More concretely, it has been evidenced that the number of circulating neutrophils, monocytes, and lymphocytes are positively correlated with BMI, body fat, and insulin resistance [38]. Moreover, the prevalence of MetS and MetS components increases white blood cell count in both men and women, which may be useful as a marker of low-grade systemic inflammation and cardiovascular disease risk [39,40]. 

The present study reveals that patients, both men and women, with MetS show a situation of high oxidative stress and a proinflammatory state with respect to non-MetS group, and this fact may contribute to the pathogenesis of MetS. Hyperglycaemia and hyperlipidaemia have been reported to increase ROS production and oxidative damage, contributing to the development of insulin resistance, altered endothelial dysfunction and energy metabolism, and the appearance of vascular complications [41,42]. The existence of an imbalance between antioxidant defence mechanisms such as CAT and SOD enzymes and the production of ROS increases the risk of oxidative stress establishment. In this sense, in several oxidative stress-related disorders, a ROS overproduction and/or a reduced capability to eliminate the ROS excess are associated with a reduction of plasma antioxidant enzyme activities, leading to oxidative damage [43]. The present results showed lower CAT and SOD activities in plasma from patients with MetS in comparison with patients without MetS. In contrast, the MPO activity was higher in MetS subjects compared to those without MetS. Similar results were previously observed, showing that recurrent implantation failure patients with MetS (RIF-MS) decreased SOD and CAT levels in comparison with a control group, whereas the MPO levels increased in the MetS group [16]. Moreover, it has been also reported that MPO activity progressively increases with obesity and MetS [44].

In addition, MDA plasma levels, a by-product of unsaturated fatty acid peroxidation [45], were higher in patients with MetS. Our findings are consistent with previous results [46] showing that the subjects with higher inflammatory status (hs-CRP ≥ 3.0 mg/L) had also significantly higher MDA levels and lower antioxidant enzymes activities. This higher MDA levels could derive from the lower plasma antioxidant enzymes and to the over active leukocytes, which are the primary source of ROS in inflammatory disorders such as MetS [47]. 

Subjects with MetS exhibited significant higher BMI and abdominal obesity, which would participate in the increased inflammatory status in these patients and, consequently, higher antioxidant protein levels and enzymatic activities in PBMCs [48]. MetS patients show a proinflammatory state with the release to the systemic circulation of inflammatory cytokines and chemoattractant mediators, and this fact can lead to an overactivation of leukocytes. This activation state has been associated with an increased NADPH oxidase and MPO activities which can contribute to the instauration of oxidative stress [49]. Altogether, this may trigger the activation of the antioxidant defenses of these PBMCs as a self-defense mechanism against the production of the highly reactive superoxide anion and hypochlorite. Specifically, the present study shows a higher significant CAT and GRd activities and protein levels in patients with MetS. 

The increased activity and level of antioxidant defences in PBMCs may derive from the situation of chronic low-grade inflammation and oxidative stress that generate continued stimuli, causing these cells to be in a pre-activated state [50]. Although to our knowledge there are no previous studies evidencing this state of pre-activation in patients with MetS, there are previous studies that corroborate the prooxidant and proinflammatory state of PBMCs in a situation of oxidative stress derived from exercise [51,52]. In fact, it has been observed that levels of antioxidant enzymes in PBMCs were induced by physical activity related to an increased oxygen consumption, allowing a better antioxidant response [51]. Moreover, the increased activity of the antioxidant enzymes in PBMCs after exercise could also be attributed to the activation of pre-existing enzymes [52]. An interesting study also evidenced that consumption of a high-saturated fatty acid diet increased the postprandial oxidative stress generated mainly in adipose tissue inducing the expression of antioxidant genes in PBMCs [53].

This study reveals that inflammatory mediators in plasma, IL-6, and TNFα, exhibited higher significant levels in patients with MetS in comparison to patients without MetS. TNFα circulates throughout the body responding to stimuli, such as infectious agents or tissue injury, altering properties of vascular endothelial cells, and regulating metabolic activities of other tissues [16,46]. The expression of IL-6 is readily induced by a variety of cytokines, lipopolysaccharide, or viral infections; for these reasons, high IL-6 levels are associated with inflammatory responses [54]. Therefore, high TNFα and IL-6 levels are also associated with MetS risk factors such as obesity, insulin resistance, and T2DM [19,55]. The high circulating levels of TNFα and IL-6 observed in MetS patients could derive from the adipose tissue expansion by adipocyte hypertrophy and hyperplasia, leading to proinflammatory cytokines (or adipokines) production. In this sense, an association between visceral/epicardial fat and chronic low-grade inflammation has been reported [56]. Therefore, subjects with MetS showed higher levels of inflammatory cytokines such as IL-6, TNFα, IL-1, IL-6, IL-10, and the chemoattractant MCP-1 in plasma and higher macrophage infiltration, in accordance with our present results [57]. Moreover, circulating immune cells can also contribute to the increased IL-6 and TNFα since a greater production of proinflammatory cytokines has been evidenced in obese respect to nonobese subjects [58]. Moreover, the induction of oxidative stress by H_2_O_2_ to PBMCs induced the expression of the toll-like receptors (TLRs) 2 and 4 and upregulated the synthesis of proinflammatory cytokines in a NF-κB-dependent pathway, reinforcing the idea of the implication of PBMCs in the metabolic inflammation [59]. 

## 5. Conclusions

The present study has evidenced the coexistence of an increased oxidative stress and a systemic proinflammatory status in patients with MetS in both genders when compared with non-MetS subjects. The systemic proinflammatory state of patients with MetS can induce the mobilization and activation of PBMCs, which is evidenced by an increase in antioxidant defense mechanisms, probably derived from a pre-activation state of immune cells.

## Figures and Tables

**Table 1:** Characteristics of participants “without Metabolic Syndrome” (n = 80) and “with Metabolic Syndrome” (n = 80) stratified by gender.

	Men		Women		ANOVA		
	Without MetS (n = 40)	With MetS (n = 40)	Without MetS (n = 40)	With MetS (n = 40)	MetS	G	MetSxG
	Mean ± SEM	Mean ± SEM	Mean ± SEM	Mean ± SEM			
Age (years)	65.7 ± 0.8	63.8 ± 0.8	66.8 ± 0.8	64.1 ± 0.5	0.003	0.335	0.612
Weight (kg)	80.0 ± 1.7 *#	93.4 ± 2.2 #	64.0 ± 1.4 *	77.1 ± 2.9	**<0.001**	**<0.001**	0.915
Height (cm)	170.0 ± 1.1 #	169.3 ± 0.9 #	156.4 ± 0.6	154.1 ± 0.9	0.102	**<0.001**	0.379
BMI (kg/m^2^)	27.6 ± 0.5 *	32.7 ± 0.7	26.1 ± 0.5 *	33.3 ± 0.8	**<0.001**	0.491	0.106
Total physical activity (MET·hour/week)	61.4 ± 3.5 ^a^	52.9 ± 6.9 ^a^	53.1 ± 2.3 ^a^	33.3 ± 4.8 ^b^	**0.027**	**<0.001**	**0.028**
Systolic blood pressure (mmHg)	138.1 ± 3.4	138.8 ± 4.7	134.7 ± 2.4	127.4 ± 4.1	0.404	0.095	0.431
Diastolic blood pressure (mmHg)	81.6 ± 1.6	75.4 ± 2.4	79.1 ± 1.4	73.2 ± 2.3	**0.003**	0.265	0.868
Glucose (mg/dL)	97.8 ± 1.5 *	120.9 ± 6.8	90.0 ± 1.2 *	108.9 ± 4.6	**<0.001**	**0.012**	0.486
Triglycerides (mg/dL)	95.0 ± 5.4 *	153.5 ± 15.4	86.0 ± 4.7 *	149.2 ± 10.4	**<0.001**	0.464	0.865
HDL-cholesterol (mg/dL)	49.95 ± 1.20 ^a^	42.9 ± 1.60 ^b^	63.4 ± 1.47 ^c^	48.7 ± 1.34 ^a^	**<0.001**	**<0.001**	**0.017**
Abdominal obesity (cm)	95.0 ± 1.5 *#	114.7 ± 1.8 #	81.0 ± 1.2 *	103.4 ± 3.2	**<0.001**	**<0.001**	0.359
WHtR	0.559 ± 0.010 *	0.659 ± 0.020	0.518 ± 0.008 *	0.671 ± 0.020	**<0.001**	**0.046**	0.505

Abbreviations: BMI: Body mass index; MET: Metabolic equivalent of task; MetS: Metabolic syndrome; SEM: Standard error media; WHtR: Waist-to-height Ratio. *MetS* effect of metabolic syndrome, *G* effect of gender, *MetS x G* interaction between metabolic syndrome and gender. Results are expressed as mean ± SEM. Statistical analysis: Two-way ANOVA, data points in bold are significant, *p* < 0.05. * Differences in means between participants without and with MetS. # Differences in means between genders. When there is an interaction, *MetS x G* exists between Metabolic syndrome and gender. Different lowercase letters reveal significant differences.

**Table 2:** Hematological parameters of participants “without Metabolic Syndrome” (n = 80) and “with Metabolic Syndrome” (n = 80) stratified by gender.

	Men		Women		ANOVA		
	Without MetS (n = 40)	With MetS (n = 40)	Without MetS (n = 40)	With MetS (n = 40)	MetS	G	MetSxG
	Mean ± SEM	Mean ± SEM	Mean ± SEM	Mean ± SEM			
Haematocrit (%)	45.8 ± 0.54 #	43.7 ± 0.78	41.5 ± 0.44	41.6 ± 0.4	0.103	**<0.001**	0.091
Erythrocytes (10^6^/mm^3^)	4.97 ± 0.06 ^a^	4.77 ± 0.09 ^a,b^	4.56 ± 0.05 ^b^	4.67 ± 0.07 ^b^	0.508	**<0.001**	**0.041**
Leukocytes (10^3^/mm^3^)	6.27 ± 0.24 *	7.66 ± 0.30	5.63 ± 0.19 *	7.22 ± 0.30	**<0.001**	**0.049**	0.637
Neutrophils (10^3^/mm^3^)	3.48 ± 0.20 *#	4.36 ± 0.21	2.72 ± 0.12 *	3.83 ± 0.19	**<0.001**	**0.001**	0.461
Lymphocytes (10^3^/mm^3^)	2.02 ± 0.08	2.27 ± 0.14	2.21 ± 0.11	2.52 ± 0.12	**0.018**	0.055	0.806
Basophils (10^3^/mm^3^)	0.036 ± 0.004 *	0.054 ± 0.005	0.040 ± 0.004	0.055 ± 0.004	**<0.001**	0.321	0.950
Monocytes (10^3^/mm^3^)	0.517 ± 0.021 *	0.678 ± 0.036	0.435 ± 0.023 *	0.596 ± 0.029	**<0.001**	**0.002**	0.840
Eosinophils (10^3^/mm^3^)	0.220 ± 0.032	0.238 ± 0.019	0.146 ± 0.012	0.217 ± 0.024	0.055	0.067	0.176

Results are expressed as mean ± SEM. Statistical analysis: Two-way ANOVA, data points in bold are significant, *p* < 0.05. *MetS* effect of metabolic syndrome, *G* effect of gender, *MetS x G* interaction between metabolic syndrome and gender. * Differences in means between participants without and with MetS. # Differences in means between genders. Different lowercase letters reveal significant differences.

**Table 3:** Plasma and peripheral blood mononuclear cells (PBMCs) enzymatic activities and protein levels of participants “without Metabolic Syndrome” (n = 80) and “with Metabolic Syndrome” (n = 80) stratified by gender.

	Men		Women		ANOVA		
	Without MetS (n = 40)	With MetS (n = 40)	Without MetS (n = 40)	With MetS (n = 40)	MetS	G	MetSxG
	Mean ± SEM	Mean ± SEM	Mean ± SEM	Mean ± SEM			
**Plasma activity**							
CAT (kat/L blood)	84.4 ± 19.5	68.3 ± 6.7	123.6 ± 28.0	83.8 ± 14.8	0.118	0.116	0.548
SOD (pkat/L sang)	660.7 ± 44.7 *	339.9 ± 23.3	689.5 ± 49.3 *	331.4 ± 16.6	**<0.001**	0.690	0.690
MPO (μkat/mL blood)	85.1 ± 25.4 *	186.4 ± 22.2	90.1 ± 26.1	163.0 ± 23.9	**<0.001**	0.614	0.478
**PBMCs activity**							
CAT (kat/10^9^ cells)	21.5 ± 4.63 *	42.2 ± 4.94	22.4 ± 3.08	29.2 ± 3.18	**0.001**	0.145	0.096
GRd (nkat/10^9^ cells)	211.5 ± 32.4 *	408.2 ± 46.0	222.3 ± 38.3 *	548.5 ± 55.7	**<0.001**	0.114	0.177
GPx (nkat/10^9^ cells)	56.0 ± 7.9	75.5 ± 5.8	54.9 ± 12.6	65.5 ± 6.3	0.059	0.496	0.590
SOD (nkat/10^9^ cells)	72.3 ± 15.9	73.1 ± 5.61	48.1 ± 9.34	68.7 ± 6.10	0.242	0.123	0.280
**PBMCs protein levels**							
CAT (%)	100.8 ± 6.6 *	159.5 ± 10.5	100.0 ± 7.1 *	130.1 ± 11.2	**<0.001**	0.200	0.229
GRd (%)	90.4 ± 12.9 *	132.6 ± 11.3	100.0 ± 11.8 *	148.4 ± 15.9	**0.001**	0.398	0.877
GPx (%)	97.5 ± 6.6	96.5 ± 8.5	100.0 ± 8.8	113.9 ± 8.8	0.477	0.336	0.497
MnSOD (%)	97.0 ± 15.2	82.7 ± 17.7	100.0 ± 12.8	84.6 ± 8.42	0.293	0.864	0.967

Abbreviations: CAT: Catalase; SOD: Superoxide dismutase; MPO: Myeloperoxidase; GRd: Glutathione reductase; GPx: Glutathione peroxidase. MnSOD: Mitochondrial antioxidant Manganese Superoxide dismutase. Statistical analysis: Two-way ANOVA, data points in bold are significant, *p* < 0.05. *MetS* effect of metabolic syndrome, *G* effect of gender, *MetS x G* interaction between metabolic syndrome and gender. Different lowercase letters reveal significant differences. Results are expressed as mean ± SEM. * Differences in means between participants without and with MetS.

## References

[1] Gami A.S., Witt B.J., Howard D.E., Erwin P.J., Gami L.A., Somers V.K., Montori V.M. (2007). Metabolic Syndrome and Risk of Incident Cardiovascular Events and Death. J. Am. Coll. Cardiol..

[2] Salas R., Bibiloni M.d.M., Ramos E., Villarreal J.Z., Pons A., Tur J.A., Sureda A. (2014). Metabolic syndrome prevalence among Northern Mexican adult population. PLoS ONE.

[3] Guallar-Castillón P., Pérez R.F., López García E., León-Muñoz L.M., Aguilera M.T., Graciani A., Gutiérrez-Fisac J.L., Banegas J.R., Rodríguez-Artalejo F. (2014). Magnitud y manejo del síndrome metabólico en España en 2008-2010: Estudio ENRICA. Rev. Española Cardiol..

[4] Beigh S.H., Jain S. (2012). Prevalence of metabolic syndrome and gender differences. Bioinformation.

[5] Vona R., Gambardella L., Cittadini C., Straface E., Pietraforte D. (2019). Biomarkers of Oxidative Stress in Metabolic Syndrome and Associated Diseases. Oxid. Med. Cell. Longev..

[6] Li S., Tan H.-Y., Wang N., Zhang Z.-J., Lao L., Wong C.-W., Feng Y. (2015). The Role of Oxidative Stress and Antioxidants in Liver Diseases. Int. J. Mol. Sci..

[7] Maritim A.C., Sanders R.A., Watkins J.B. (2003). Diabetes, oxidative stress, and antioxidants: A review. J. Biochem. Mol. Toxicol..

[8] Bekkouche L., Bouchenak M., Malaisse W.J., Yahia D.A. (2014). The mediterranean diet adoption improves metabolic, oxidative, and inflammatory abnormalities in algerian metabolic syndrome patients. Horm. Metab. Res..

[9] Stocker R., Keaney J.F. (2004). Role of Oxidative Modifications in Atherosclerosis. Physiol. Rev..

[10] Grandl G., Wolfrum C. (2018). Hemostasis, endothelial stress, inflammation, and the metabolic syndrome. Semin. Immunopathol..

[11] Lackey D.E., Olefsky J.M. (2016). Regulation of metabolism by the innate immune system. Nat. Rev. Endocrinol..

[12] Saltiel A.R., Olefsky J.M. (2017). Inflammatory mechanisms linking obesity and metabolic disease. J. Clin. Investig..

[13] Zgheib C., Xu J., Liechty K.W. (2014). Targeting Inflammatory Cytokines and Extracellular Matrix Composition to Promote Wound Regeneration. Adv. Wound Care.

[14] Mansyur M.A., Bakri S., Patellongi I.J., Rahman I.A. (2020). The association between metabolic syndrome components, low-grade systemic inflammation and insulin resistance in non-diabetic Indonesian adolescent male. Clin. Nutr. ESPEN.

[15] Welty F.K., Alfaddagh A., Elajami T.K. (2016). Targeting inflammation in metabolic syndrome. HHS Public Access.

[16] Sheikhansari G., Soltani-Zangbar M.S., Pourmoghadam Z., Kamrani A., Azizi R., Aghebati-Maleki L., Danaii S., Koushaeian L., Hojat-Farsangi M., Yousefi M. (2019). Oxidative stress, inflammatory settings, and microRNA regulation in the recurrent implantation failure patients with metabolic syndrome. Am. J. Reprod. Immunol..

[17] Savaş E.M., Oğuz S.H., Samadi A., Yılmaz Işıkhan S., Ünlütürk U., Lay İ., Gürlek A. (2020). Apoptosis Inhibitor of Macrophage, Monocyte Chemotactic Protein-1, and C-Reactive Protein Levels Are Increased in Patients with Metabolic Syndrome: A Pilot Study. Metab. Syndr. Relat. Disord..

[18] Nishimura S., Manabe I., Nagasaki M., Eto K., Yamashita H., Ohsugi M., Otsu M., Hara K., Ueki K., Sugiura S. (2009). CD8+ effector T cells contribute to macrophage recruitment and adipose tissue inflammation in obesity. Nat. Med..

[19] Esser N., Legrand-Poels S., Piette J., Scheen A.J., Paquot N. (2014). Inflammation as a link between obesity, metabolic syndrome and type 2 diabetes. Diabetes Res. Clin. Pract..

[20] Richard C., Wadowski M., Goruk S., Cameron L., Sharma A.M., Field C.J. (2017). Individuals with obesity and type 2 diabetes have additional immune dysfunction compared with obese individuals who are metabolically healthy. BMJ Open Diabetes Res. Care.

[21] Morohoshi M., Fujisawa K., Uchimura I., Numano F. (2006). The Effect of Glucose and Advanced Glycosylation End Products on IL-6 Production by Human Monocytes. Ann. N. Y. Acad. Sci..

[22] Reinhold D., Ansorge S. (1996). Elevated Glucose Levels Stimulate Transforming Growth Factor-β1 (TGF-β1), Suppress Interleukin IL-2, IL-6 and IL-10 Production and DNA Synthesis in Peripheral Blood Mononuclear Cells. Horm. Metab. Res..

[23] Alberti K.G.M.M., Eckel R.H., Grundy S.M., Zimmet P.Z., Cleeman J.I., Donato K.A., Fruchart J.-C., James W.P.T., Loria C.M., Smith S.C. (2009). Harmonizing the Metabolic Syndrome: A Joint Interim Statement of the International Diabetes Federation Task Force on Epidemiology and Prevention; National Heart, Lung, and Blood Institute; American Heart Association; World Heart Federation; International Atherosclerosis Society; and International Association for the Study of Obesity. Circulation.

[24] Ainsworth B.E., Haskell W.L., Leon A.S., Jacobs D.R., Montoye H.J., Sallis J.F., Paffenbarger R.S. (1993). Compendium of Physical Activities: Classification of energy costs of human physical activities. Med. Sci. Sport. Exerc..

[25] Bøyum A. (1964). Separation of White Blood Cells. Nature.

[26] Mestre-Alfaro A., Ferrer M.D., Sureda A., Tauler P., Martínez E., Bibiloni M.M., Micol V., Tur J.A., Pons A. (2011). Phytoestrogens enhance antioxidant enzymes after swimming exercise and modulate sex hormone plasma levels in female swimmers. Eur. J. Appl. Physiol..

[27] Busquets-Cortés C., Capó X., Martorell M., Tur J.A., Sureda A., Pons A. (2016). Training Enhances Immune Cells Mitochondrial Biosynthesis, Fission, Fusion, and Their Antioxidant Capabilities Synergistically with Dietary Docosahexaenoic Supplementation. Oxid. Med. Cell. Longev..

[28] Aebi H. (1984). Catalase in vitro. Methods Enzymol..

[29] McCord J.M., Fridovich I. (1969). Superoxide dismutase. An enzymic function for erythrocuprein (hemocuprein). J. Biol. Chem..

[30] Bergmayer H.U. (1963). Glutathione reductase. Methods of Enzymatic Analysis. Starch - Stärke.

[31] Flohé L., Günzler W.A. (1984). Assays of glutathione peroxidase. Methods Enzymol..

[32] Capeillère-Blandin C. (1998). Oxidation of guaiacol by myeloperoxidase: A two-electron-oxidized guaiacol transient species as a mediator of NADPH oxidation. Biochem. J..

[33] Zimmet P.Z., McCarty D.J., de Courten M.P. (1997). The global epidemiology of non-insulin-dependent diabetes mellitus and the metabolic syndrome. J. Diabetes Complicat..

[34] Bulló M., Casas-Agustench P., Amigó-Correig P., Aranceta J., Salas-Salvadó J. (2007). Inflammation, obesity and comorbidities: The role of diet. Public Health Nutr..

[35] de Eguilaz M.H.R., Batlle M.A., de Morentin B.M., San-Cristóbal R., Pérez-Díez S., Navas-Carretero S., Martínez J.A. (2016). Cambios alimentarios y de estilo de vida como estrategia en la prevención del síndrome metabólico y la diabetes mellitus tipo 2: Hitos y perspectivas Alimentary and lifestyle changes as a strategy in the prevention. An. Sist. Sanit. Navar.

[36] Nihi M.M., Manfro R.C., Martins C., Suliman M., Murayama Y., Riella M.C., Lindholm B., Nascimento M.M. (2010). do [Association between body fat, inflammation and oxidative stress in hemodialysis]. J. Bras. Nefrol..

[37] Sureda A., Bibiloni M., Julibert A., Bouzas C., Argelich E., Llompart I., Pons A., Tur J. (2018). Adherence to the Mediterranean Diet and Inflammatory Markers. Nutrients.

[38] Ryder E., Diez-Ewald M., Mosquera J., Fernández E., Pedreañez A., Vargas R., Peña C., Fernández N. (2014). Association of obesity with leukocyte count in obese individuals without metabolic syndrome. Diabetes Metab. Syndr. Clin. Res. Rev..

[39] Oda E., Kawai R. (2009). The Prevalence of Metabolic Syndrome and Diabetes Increases through the Quartiles of White Blood Cell Count in Japanese Men and Women. Intern. Med..

[40] Li P.-F., Chen J.-S., Chang J.-B., Chang H.-W., Wu C.-Z., Chuang T.-J., Huang C.-L., Pei D., Hsieh C.-H., Chen Y.-L. (2016). Association of complete blood cell counts with metabolic syndrome in an elderly population. BMC Geriatr..

[41] Oguntibeju O.O. (2019). Type 2 diabetes mellitus, oxidative stress and inflammation: Examining the links. Int. J. Physiol. Pathophysiol. Pharmacol..

[42] Okon E.B., Chung A.W.Y., Zhang H., Laher I., van Breemen C. (2007). Hyperglycemia and hyperlipidemia are associated with endothelial dysfunction during the development of type 2 diabetes. Can. J. Physiol. Pharmacol..

[43] Rizzo A., Roscino M., Binetti F., Sciorsci R. (2012). Roles of Reactive Oxygen Species in Female Reproduction. Reprod. Domest. Anim..

[44] Sladoje D.P., Kisić B., Mirić D. (2017). The Monitoring of Protein Markers of Inflammation and Serum Lipid Concentration in Obese Subjects with Metabolic Syndrome. J. Med. Biochem..

[45] Capó X., Martorell M., Sureda A., Riera J., Drobnic F., Tur J.A., Pons A. (2016). Effects of Almond- and Olive Oil-Based Docosahexaenoic- and Vitamin E-Enriched Beverage Dietary Supplementation on Inflammation Associated to Exercise and Age. Nutrients.

[46] Chen S.J., Yen C.H., Huang Y.C., Lee B.J., Hsia S., Lin P.T. (2012). Relationships between Inflammation, Adiponectin, and Oxidative Stress in Metabolic Syndrome. PLoS ONE.

[47] Wu S.-S., Kor C.-T., Chen T.-Y., Liu K.-H., Shih K.-L., Su W.-W., Wu H.-M. (2019). Relationships between Serum Uric Acid, Malondialdehyde Levels, and Carotid Intima-Media Thickness in the Patients with Metabolic Syndrome. Oxid. Med. Cell. Longev..

[48] Ferrer M.D., Tauler P., Sureda A., Tur J.A., Pons A. (2009). Antioxidant regulatory mechanisms in neutrophils and lymphocytes after intense exercise. J. Sports Sci..

[49] Hutcheson R., Rocic P. (2012). The Metabolic Syndrome, Oxidative Stress, Environment, and Cardiovascular Disease: The Great Exploration. Exp. Diabetes Res..

[50] Ladeiras-Lopes R., Teixeira P., Azevedo A., Leite-Moreira A., Bettencourt N., Fontes-Carvalho R. (2019). Metabolic syndrome severity score is associated with diastolic dysfunction and low-grade inflammation in a community-based cohort. Eur. J. Prev. Cardiol..

[51] Busquets-Cortés C., Capó X., del Mar Bibiloni M., Martorell M., Ferrer M.D., Argelich E., Bouzas C., Carreres S., Tur J.A., Pons A. (2018). Peripheral blood mononuclear cells antioxidant adaptations to regular physical activity in elderly people. Nutrients.

[52] Capó X., Martorell M., Sureda A., Llompart I., Tur J.A., Pons A. (2014). Diet supplementation with DHA-enriched food in football players during training season enhances the mitochondrial antioxidant capabilities in blood mononuclear cells. Eur. J. Nutr..

[53] Camargo A., Peña-Orihuela P., Rangel-Zúñiga O.A., Pérez-Martínez P., Delgado-Lista J., Cruz-Teno C., Marín C., Tinahones F., Malagón M.M., Roche H.M. (2014). Peripheral blood mononuclear cells as in vivo model for dietary intervention induced systemic oxidative stress. Food Chem. Toxicol..

[54] Tanaka T., Narazaki M., Kishimoto T. (2014). IL-6 in inflammation, immunity, and disease. Cold Spring Harb. Perspect. Biol..

[55] Rajendran P., Rengarajan T., Thangavel J., Nishigaki Y., Sakthisekaran D., Sethi G., Nishigaki I. (2013). The vascular endothelium and human diseases. Int. J. Biol. Sci..

[56] Carbone F., Lattanzio M.S., Minetti S., Ansaldo A.M., Ferrara D., Molina-Molina E., Belfiore A., Elia E., Pugliese S., Palmieri V.O. (2019). Circulating CRP Levels Are Associated with Epicardial and Visceral Fat Depots in Women with Metabolic Syndrome Criteria. Int. J. Mol. Sci..

[57] Jayarathne S., Koboziev I., Park O.H., Oldewage-Theron W., Shen C.L., Moustaid-Moussa N. (2017). Anti-Inflammatory and Anti-Obesity Properties of Food Bioactive Components: Effects on Adipose Tissue. Prev. Nutr. Food Sci..

[58] Sirota P., Hadi E., Djaldetti M., Bessler H. (2015). Difference in inflammatory cytokine production by mononuclear cells from obese and non-obese schizophrenic patients. Acta Psychiatr. Scand..

[59] Akhter N., Madhoun A., Arefanian H., Wilson A., Kochumon S., Thomas R., Shenouda S., Al-Mulla F., Ahmad R., Sindhu S. (2019). Oxidative Stress Induces Expression of the Toll-Like Receptors (TLRs) 2 and 4 in the Human Peripheral Blood Mononuclear Cells: Implications for Metabolic Inflammation. Cell. Physiol. Biochem..

